# Supplementary figures and images for: Age-associated changes in lineage composition of the enteric nervous system regulate gut health and disease
Source: eLife. 2023 Dec 18;12:RP88051. doi: 10.7554/eLife.88051 (PMC10727506; doi:10.7554/eLife.88051)

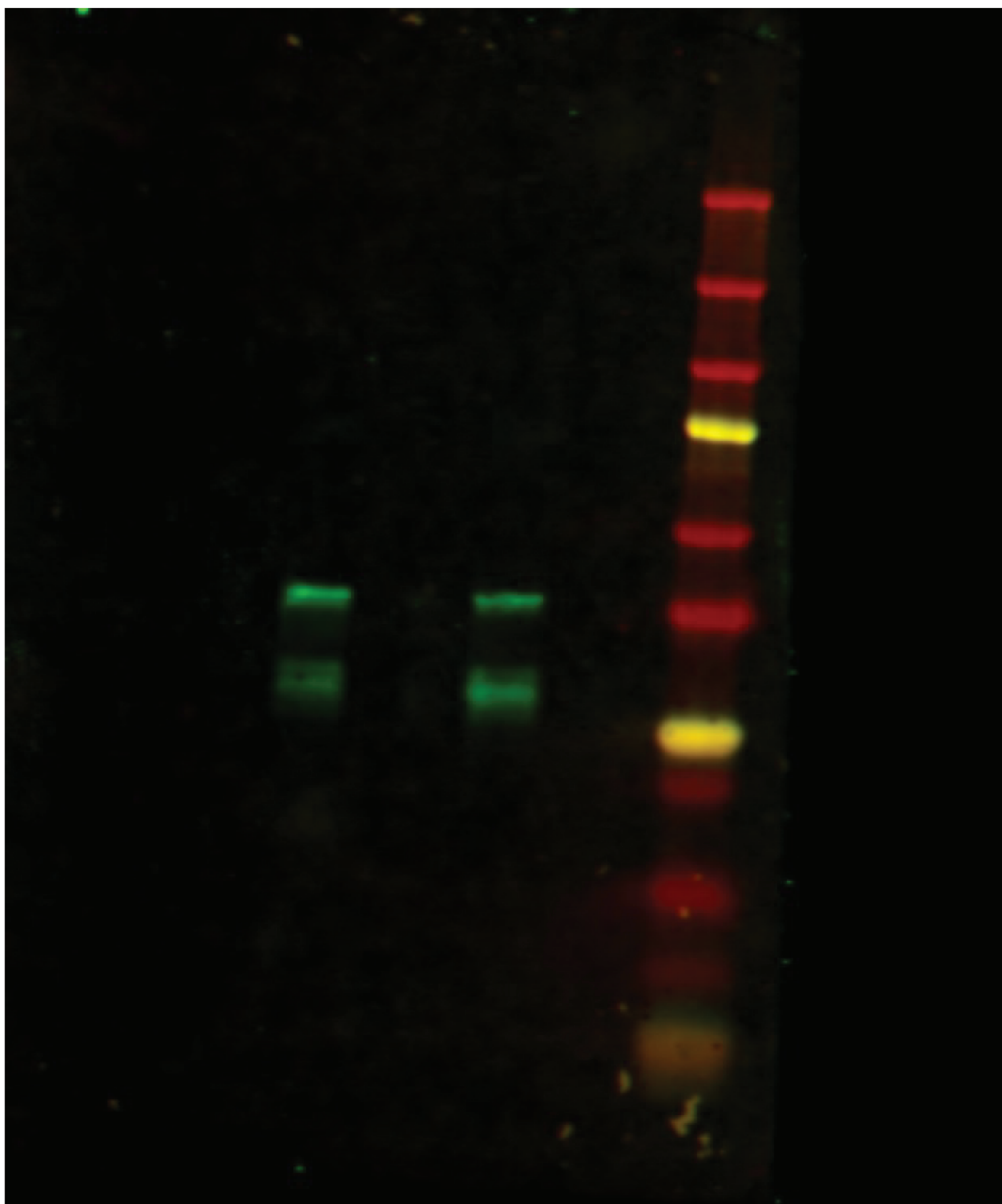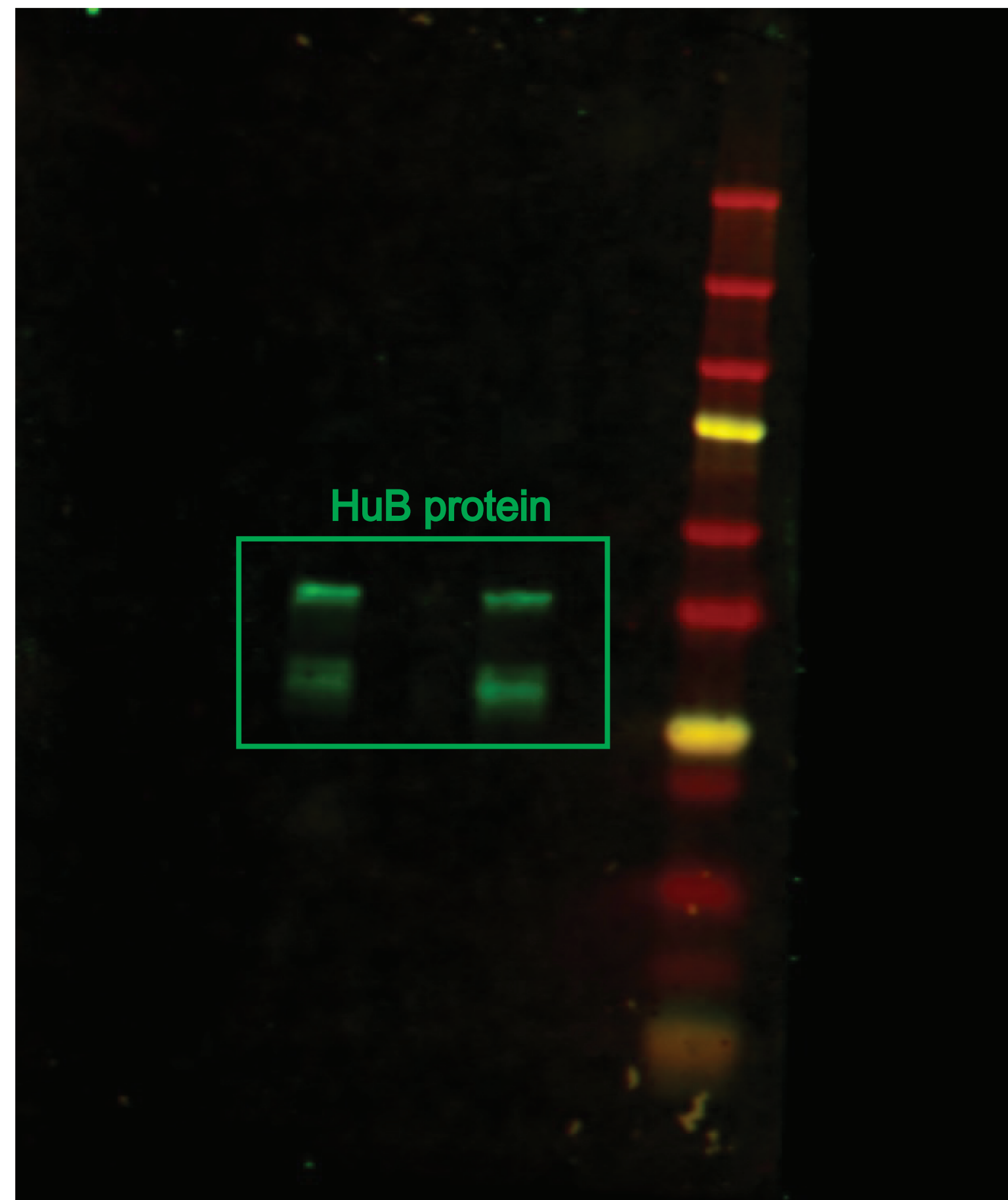

Supplement: Figure 1—figure supplement 1—source data 1. [file elife-88051-fig1-figsupp1-data1.pdf]

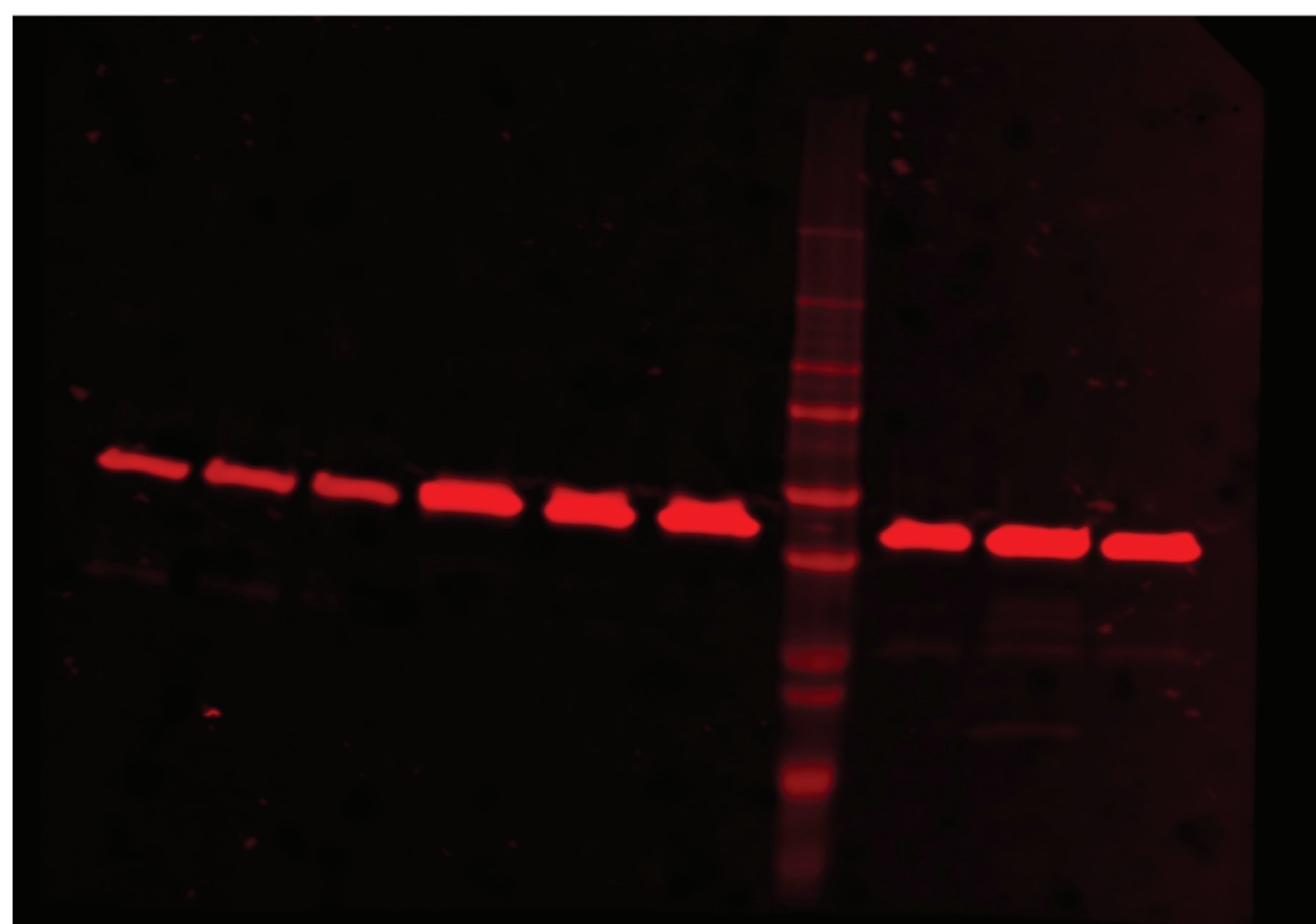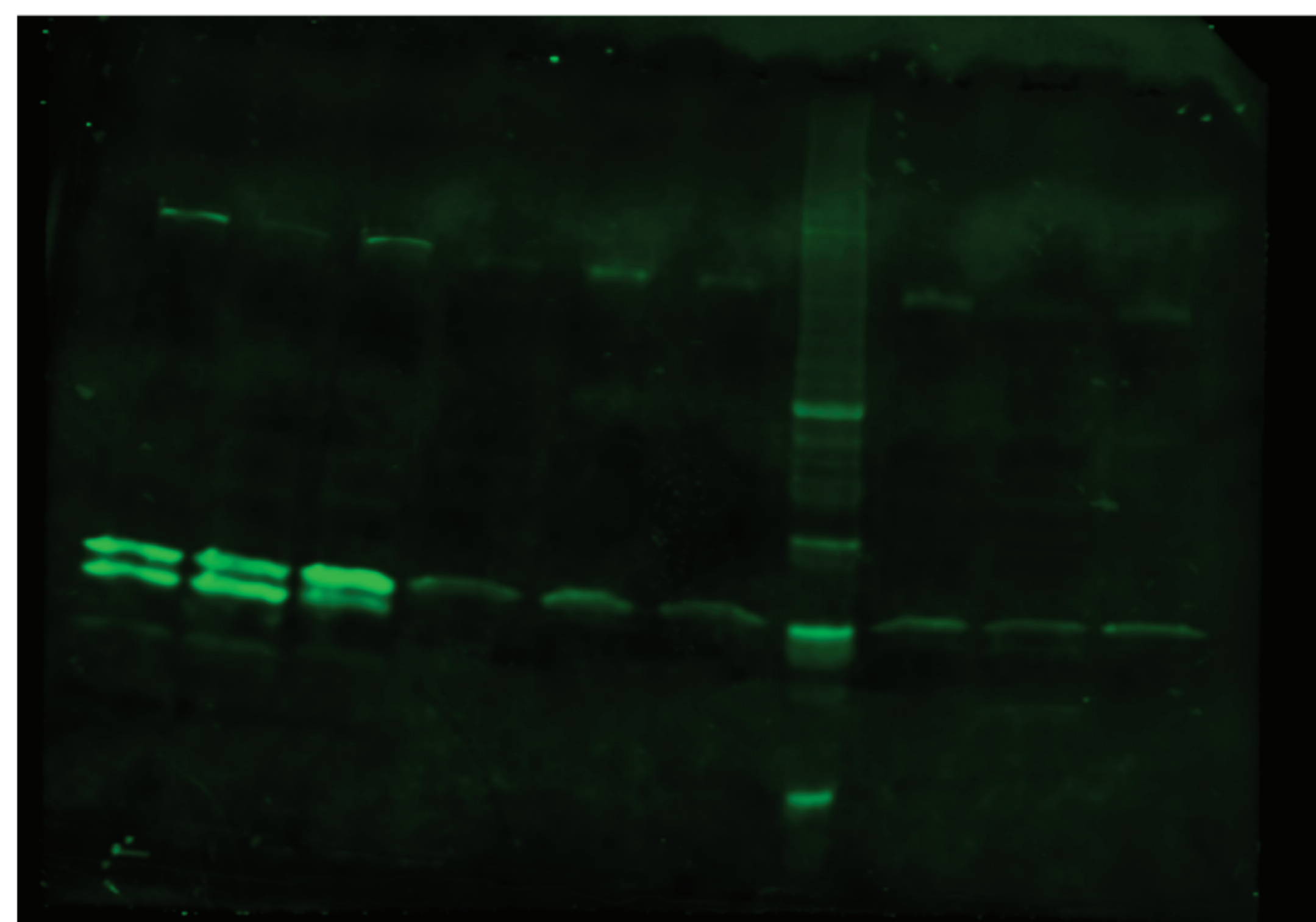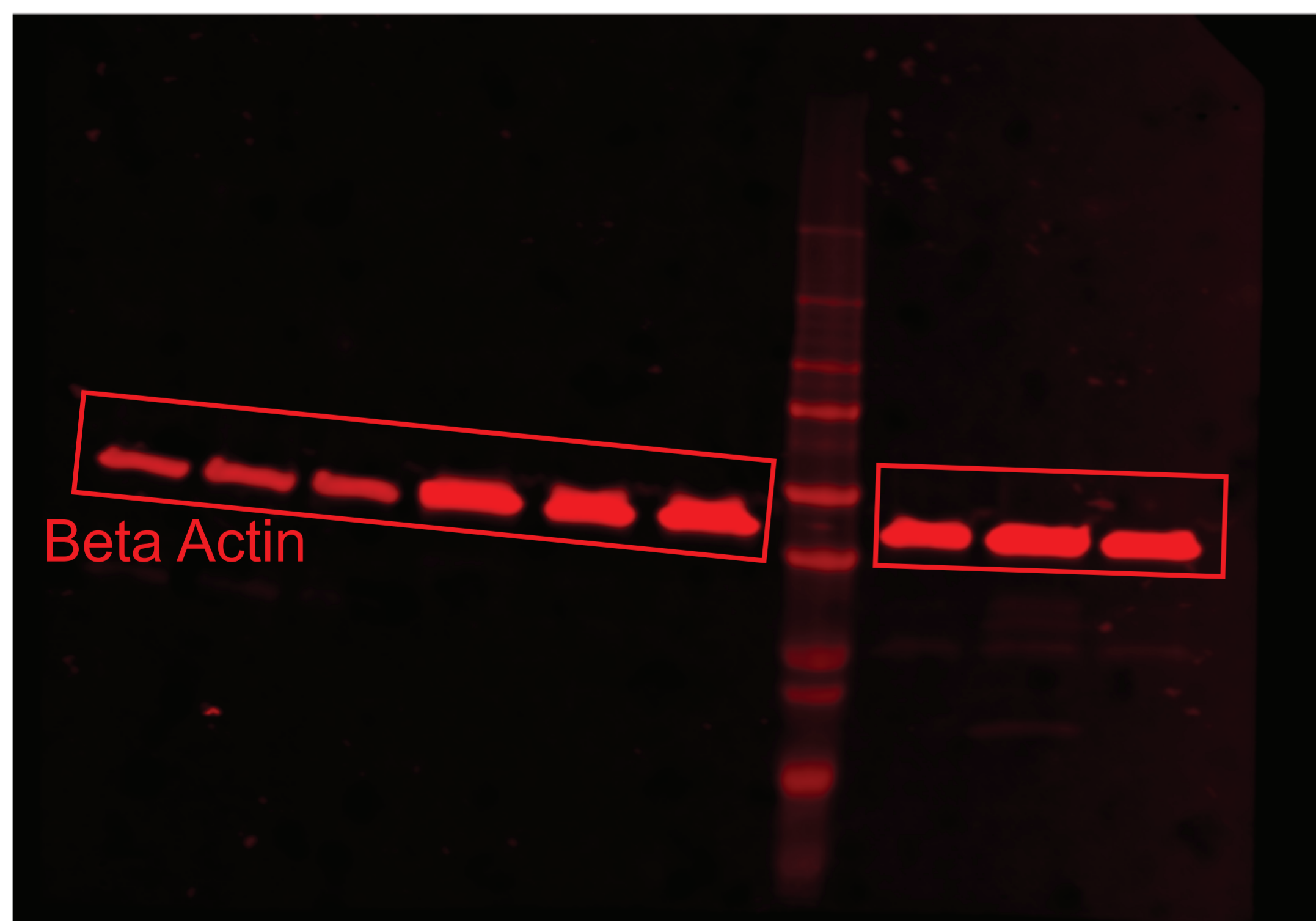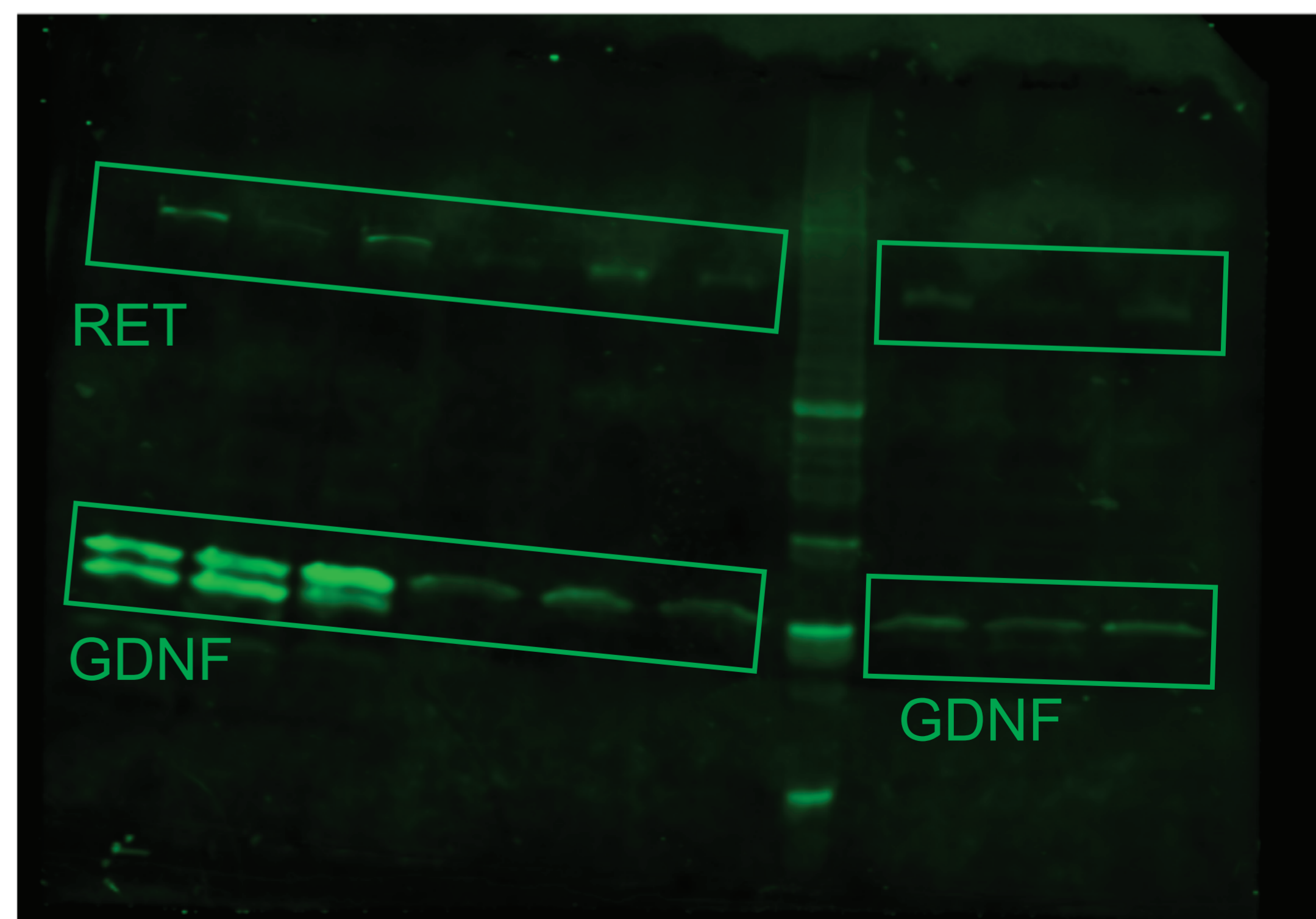

Supplement: Figure 10—source data 1. [file elife-88051-fig10-data1.pdf]

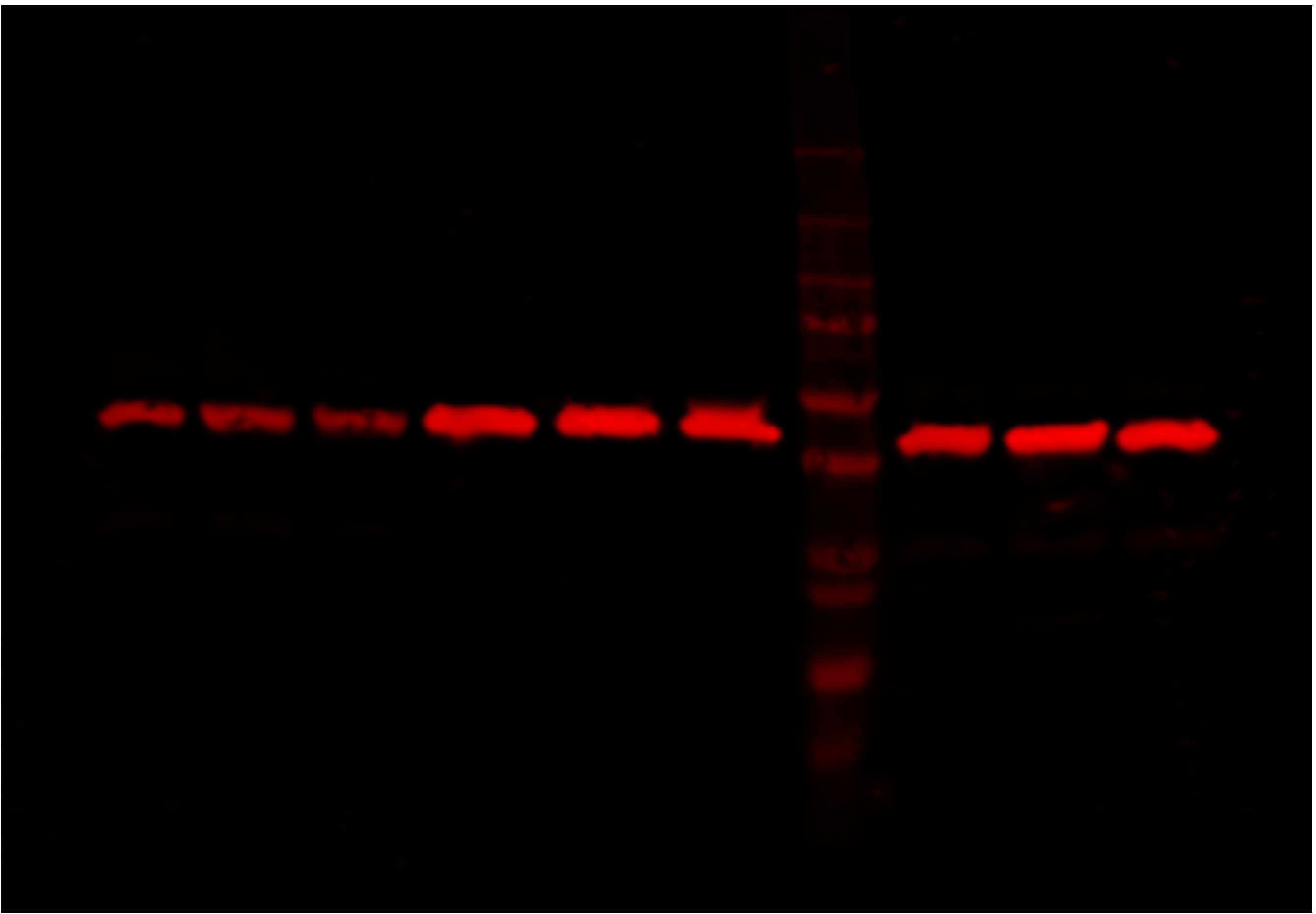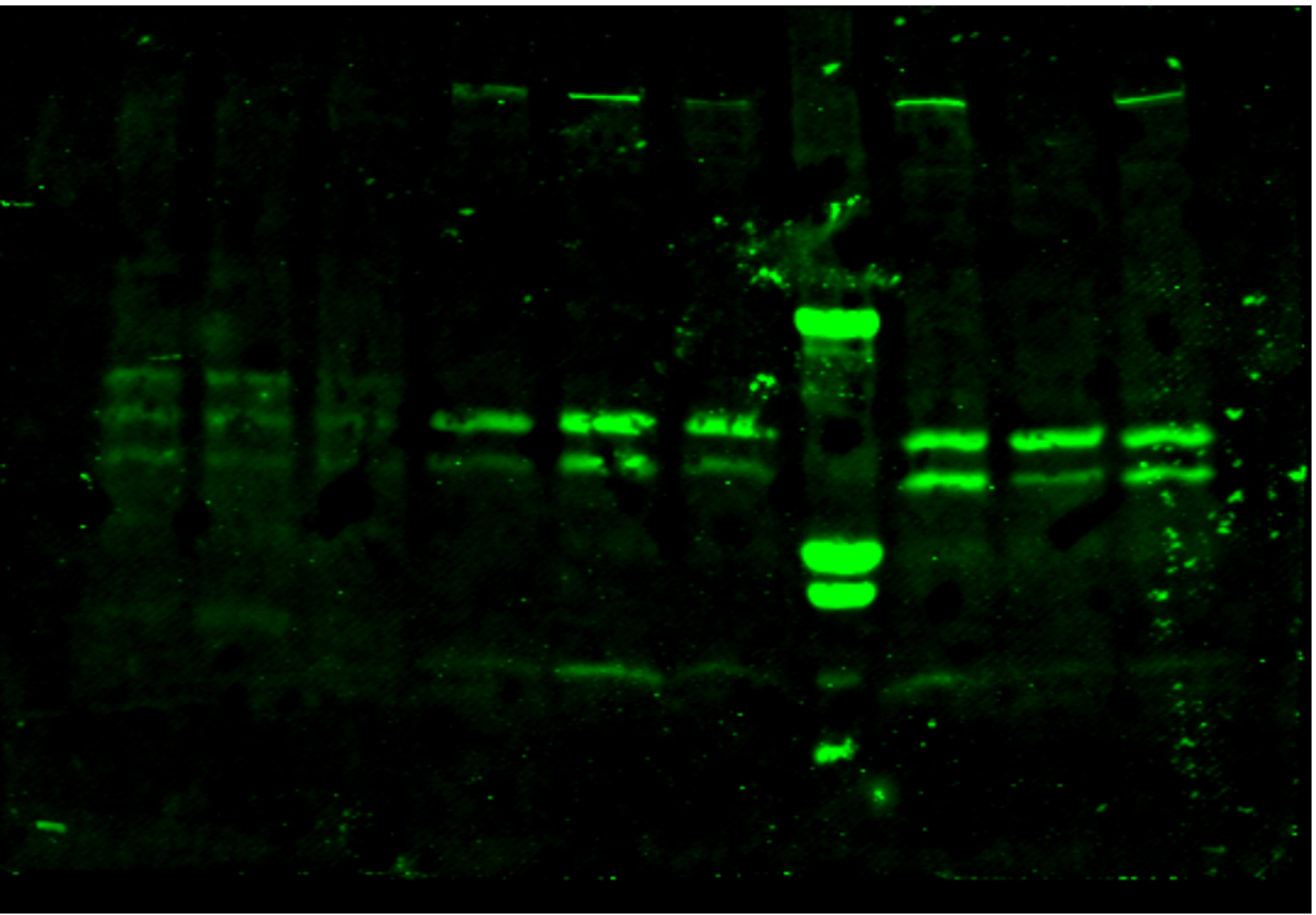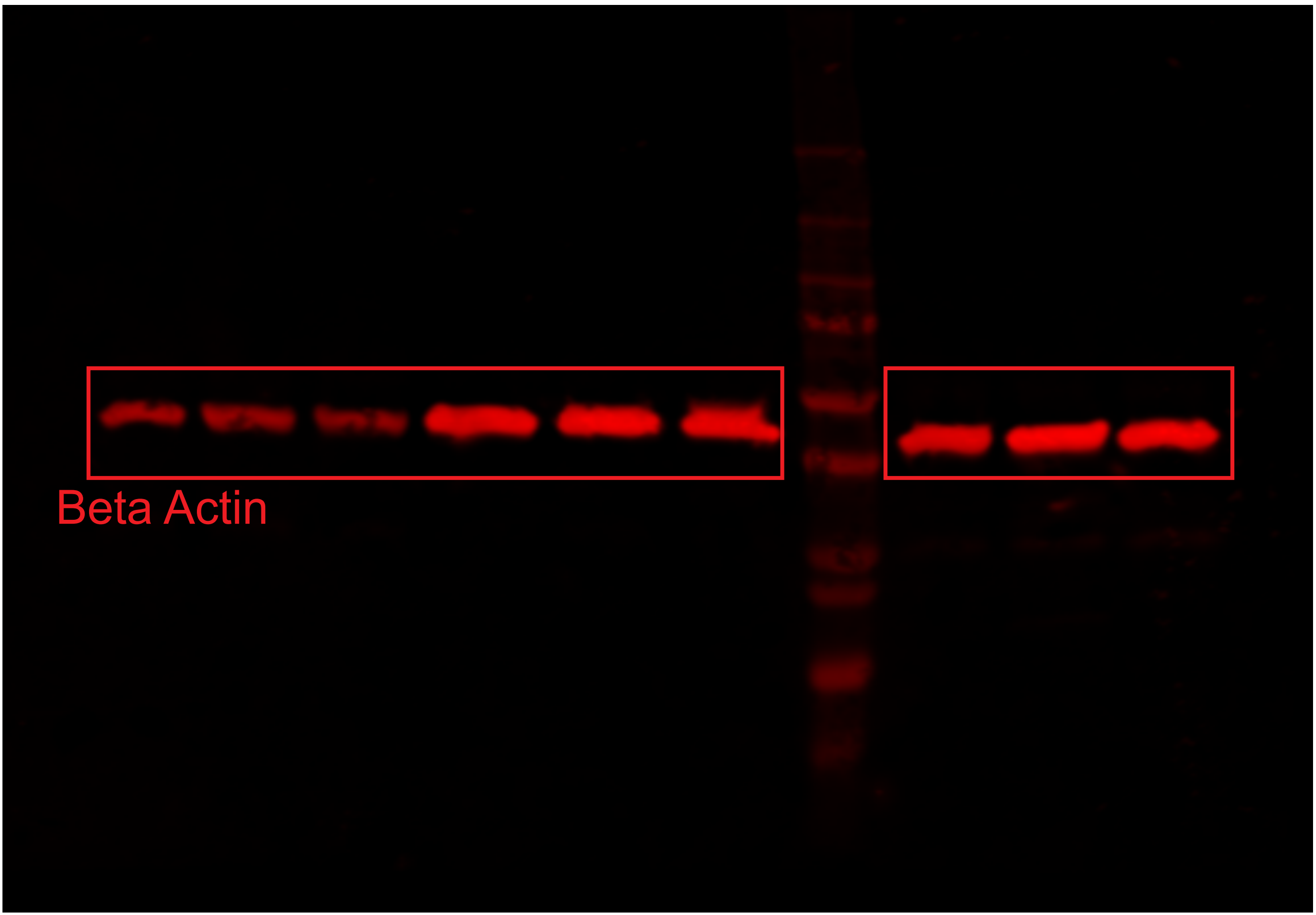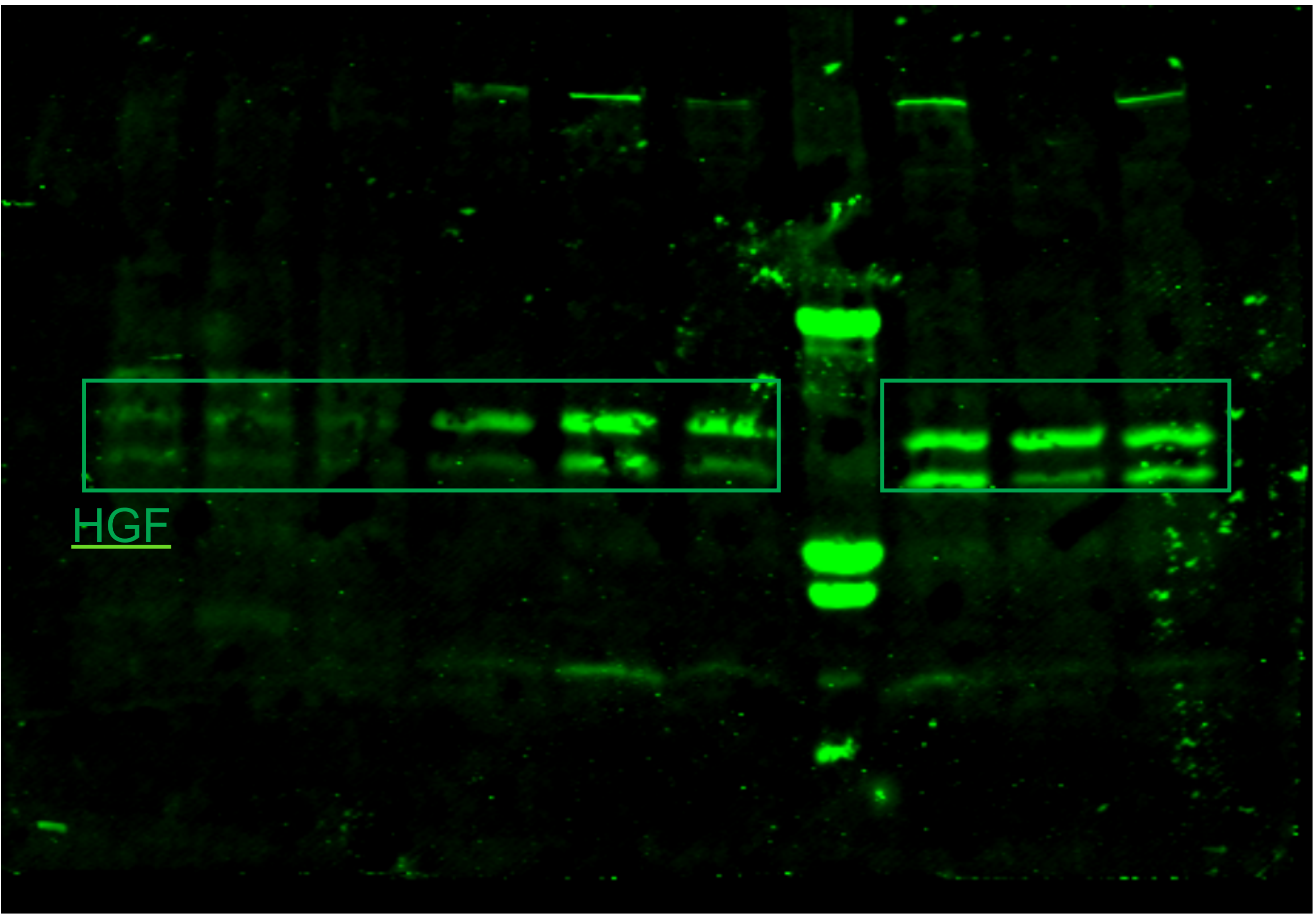

Supplement: Figure 10—source data 2. [file elife-88051-fig10-data2.pdf]
