# Supplementary material for: Age-associated changes in lineage composition of the enteric nervous system regulate gut health and disease
Source: eLife. 2023 Dec 18;12:RP88051. doi: 10.7554/eLife.88051 (PMC10727506; doi:10.7554/eLife.88051)
Supplement: Figure 4—source data 1. — Highlighted genes between MENs clusters from our data and data from May-Zhang et al and from Drokhlyansky et al show similar gene expression profiles between the clusters. [file elife-88051-fig4-data1.pdf]

Figure 4-figure supplement 4

Calcb+ cluster corresponding to Ileum cluster in May-Zhang et al. which expresses Calcb, Calb2, Nmu, Pcdh10, Cysltr2

Gene	FDR	summary.logFC
Zfp804a	0	5.314372906
Pcdh10	0	2.727131014
Gpr149	0	2.928565459
Necab1	0	3.126115371
Grin3a	0	2.477871618
Fam19a1	0	4.658451729
Ano2	0	3.443066822
Ntrk3	0	4.380099903
Cntn5	0	6.308342607
Ccbe1	0	3.076522552
Zbtb7c	0	2.656109043
Astn2	0	3.366649926
Dapk2	0	1.703870376
Nrxn3	0	5.160806272
Dgkg	0	4.15397884
Galr1	0	1.673088209
Nmu	0	3.521779106
Hspb8	1.42E-236	1.244433448
Cpne4	0	4.163433255
Tmeff2	0	3.49539549
Myl1	0	3.653772641
Calcb	0	3.200679451
Cysltr2	0	2.487957211
Clstn2	0	2.655215539
Robo1	1.98E-297	1.9960685
Hunk	0	1.918886092
Snhg11	0	2.098574269
Aff2	0	2.087576143
Otud7a	3.38E-263	2.545208984
Pcdh9	0	4.216229123
Htr3b	0	1.553388667
Scn11a	0	1.908976823
Iqgap2	0	1.75599309
Rbfox1	0	4.70241843
Tcf7l2	0	2.84136652
Tbx2	0	1.871954414
Robo2	0	4.088780154
Kcnt2	0	3.926721104
Srrm4	2.23E-261	2.303709088
Plxna4	0	3.31892195
Sgcz	0	4.739595343
9530059O14	0	3.600146025
Sox4	7.89E-259	1.211156367
Hcn1	8.400165280	1.861283774
Scube1	0	2.077291022
Boc	5.82E-199	0.854637195
Cbln2	0	2.229769856
Kcnn3	0	1.899022238
Rph3a	0	1.741082515
Cpne8	0	2.360533782
Calcrl	1.126952401	1.788279732
Lingo2	0	4.714175039
Slc4a4	0	2.575716085
Cux2	0	2.614397251
Chat	1.62E-252	1.075493057
Htr3a	0	1.82434275
Hs3st2	1.183691861	1.564650584
Cdh8	0.00E+00	2.544193879
Gm45881	0	2.045509731
Cdh6	0	2.996825969
Gm21847	9.83E-301	2.668713923
Frmd4b	0	2.260822381
Ndrg4	5.28E-172	1.033574626
Efr3a	0	2.922824682
Syt2	0	1.996197662
Pak7	2.65E-280	1.50563649
Lhfpl2	0	1.681014338
Adgrg6	0	3.290333725
Spock3	0	3.61350554
Smad6	3.00E-194	0.829179685
Zeb2	1.58E-304	2.305509194
Hs3st5	0	2.356357004
Kcnh1	1.71E-232	1.145753317
Scn9a	0	1.985971133
Ppp3ca	0	2.635476275
Agrn	0	2.149338255
Cntnap2	0	3.976062671
Ngfr	0	2.251817207
Tnr	0	2.255094218
Mcf2l	6.65E-174	0.813444301
Kcnb2	0	4.170860912
Gpr85	0	1.49922453
Nell1	0	2.464621478
Arhgef3	0	1.961289599
Syt15	3.73E-275	1.216092892
Lsamp	7.25E-253	2.548136091
5330434G04	4.84E-185	2.358912068
Ptger3	1.23E-256	1.370213397
1700111E14	4.26E-258	1.561674061
Mgat4c	0	3.086139006
Fgf14	0	3.456636428
Mafg	2.98E-74	1.352690002
Kif26b	0	2.174461286
Plppr5	1.60E-265	1.61715395
Srrm3	5.65E-165	1.711562875
Hmbox1	7.78E-79	0.6148506
Rbfox3	1.95E-259	1.230611205
Ncam2	0	2.906633451
Slc8a1	0	2.937485628

Calcb+ cluster corresponding to Ileum cluster in May-Zhang et al. which expresses Calcb, Calb2, Sst

Gene	FDR	summary.logFC
Nlgn1	1.56E-227	4.503778932
Plxna4	5.40E-170	3.513261638
Pcdh15	6.75E-269	4.552606891
Nrg3	9.23E-246	4.051012028
Eml6	1.02E-166	3.739252675
Ryr2	4.15E-122	2.724692337
Nrxn3	1.20E-156	4.845162261
Rbfox1	0.00E+00	4.833341666
Sst	6.45E-135	5.862426929
Chsy3	4.53E-122	3.602794203
Gria2	3.32E-126	3.083755369
Ptprd	3.17E-139	2.906173926
Pde4b	3.69E-210	3.947198917
Fam19a2	8.41E-100	3.227261386
Mdga2	2.01E-132	3.667573403
Snhg11	6.24E-112	4.344115315
Ralyl	1.59E-128	3.809356707
Bnc2	1.58E-137	3.131344621
Robo1	8.43E-84	2.405353602
Nrxn1	1.51E-150	4.662080188
Pcsk2	5.79E-169	3.258681935
Syn2	1.17E-132	3.619085064
Ntrk3	1.05E-108	2.657539356
Scube1	1.03E-95	2.265338351
Man2a1	9.20E-139	3.375536511
Kcnn2	7.39E-85	2.418320757
Fam19a1	1.79E-147	3.622699285
Nell1	3.21E-100	2.793128177
Galntl6	1.72E-109	3.815091788
Sgcd	5.24E-120	2.88253651
Pde4d	1.18E-97	2.285404689
Slc8a1	1.08E-180	3.289543486
Tcf4	1.52E-159	2.592805317
Pak3	2.89E-91	2.580345128
Kcnma1	1.27E-136	2.945051283
Adgrb3	5.63E-143	4.396153392
Unc5c	2.09E-114	3.291001111
Cpne4	4.42E-81	2.730862875
Meg3	7.04E-124	4.308953415
Snap25	4.22E-135	3.917101813
Syt1	7.05E-140	4.346683131
Grik4	8.53E-115	2.35016852
Ptprn2	1.75E-115	3.282060606
Arhgap26	1.46E-133	2.381300441
Bcl2	1.53E-91	2.27434062
Ppm1h	6.46E-139	3.466729684
Fgf14	3.30E-126	4.330212767
Gm12216	9.54E-78	1.937925878
Camk2a	1.73E-88	2.352765766
Fgf13	5.32E-123	3.932808506
Ppp3ca	1.96E-101	2.03501536
Adamts9	1.24E-60	1.993937158
Calcb	4.08E-76	2.288867891
Pcbp3	1.17E-136	3.893280923
Dlgap2	1.21E-96	2.470978745
Nkain2	1.01E-107	2.761261609
Chat	2.41E-66	1.619335139
Gfra2	4.04E-106	2.379843878
Pde10a	7.37E-115	2.924056301
Ptprr	1.01E-130	3.346248491
Plod2	7.61E-111	2.338515945
Hs6st3	2.71E-78	3.027568338
Rbms3	2.24E-117	1.826125797
Ccbe1	2.81E-69	1.972703173
Trpm3	3.63E-112	3.250658339
Frmd4a	4.22E-106	3.509014084
Galnt17	5.29E-90	2.14675017
Itpr1	1.09E-85	2.066072139
Srrm4	1.17E-82	2.302220849
Slc7a14	2.27E-93	2.415887276
Sema6d	4.44E-99	2.246929266
Pcdh7	8.46E-105	3.303407952
Nwd2	6.48E-47	1.597139988
Slit3	2.09E-90	2.828190275
Asic2	3.26E-63	3.678490279
Stxbp5l	1.22E-98	2.855884955
Smarca2	2.65E-97	2.286413106
Ptprt	3.14E-93	2.816471863
Dpyd	1.72E-91	3.059193551
Tln2	1.49E-97	2.183191767
Satb2	1.74E-60	2.016803391
Tshz2	5.54E-95	2.764995869
Plcxd3	4.17E-89	2.590078205
Tcf7l2	1.76E-95	2.141154837
Caln1	9.30E-64	2.04008457
Dgki	6.15E-121	3.238923425
Syt17	5.83E-76	1.809205079
9530059O14	5.05E-74	2.430525524
Unc79	7.38E-102	2.530692527
Pvt1	8.90E-90	2.163637608
Mirg	4.73E-63	1.711809423
Ret	1.04E-68	1.699484732
Nrg1	5.72E-62	3.678288908
Myt1l	1.56E-120	3.008757293
Mapk10	9.27E-117	2.871225578
Gm15398	4.78E-73	2.683512094
Arhgef28	5.64E-99	2.170007537
Zbtb20	6.81E-94	1.566902687
Dscam	2.70E-101	2.808126988

MENs-like cluster corresponding to Ileum cluster in May-Zhang et al. which expresses Upk3b and Igfbp6

Gene	FDR	summary.logFC
Abi1	4.98E-36	3.316005072
Dcn	2.08E-52	5.091738535
Col3a1	7.95E-31	3.039859427
C3	1.11E-38	4.827731701
Slpi	2.75E-31	4.598725251
Lrrn4	7.45E-21	2.797813295
S100a6	9.36E-29	3.230324987
Upk3b	1.90E-26	3.101085936
Gpm6a	1.13E-24	3.730157809
Sparc	1.37E-28	3.316214748
Meg3	3.89E-23	2.952545493
Igfbp6	4.99E-26	3.61531349
Agap1	4.03E-21	2.507795342
Pbx1	4.47E-25	2.530783608
Efemp1	2.67E-22	2.915648257
Gas6	2.54E-20	2.742900314
Muc16	2.43E-21	3.168081514
Gas1	2.13E-23	2.708510446
Dpp4	4.03E-21	2.876742349
Cobll1	1.10E-20	2.231586143
Rarres2	1.64E-20	3.226437143
Myo1d	6.05E-21	2.158327438
Tbce	1.69E-12	3.139158995
Il6st	6.05E-21	2.339891442
Nfib	7.62E-23	2.57202827
Crip1	7.17E-19	2.764477578
Sema5a	1.97E-19	2.852546212
Fmo2	1.80E-18	3.253371192
Ezr	2.84E-20	2.166036039
Ahnak	6.05E-21	2.714253195
Bnc2	6.54E-20	3.061680372
Krt19	6.15E-20	2.380492921
Crim1	3.00E-20	2.434254471
Plxna4	2.07E-19	2.707560817
Sox6	3.93E-19	2.803585619
Aebp1	1.57E-19	2.548178022
Cfh	9.08E-18	2.422456138
Col1a1	2.72E-13	2.033502612
Wt1	2.03E-17	2.196828568
Ccdc171	1.09E-14	2.022034827
Cd2ap	2.30E-18	2.290803108
Arhgap29	3.72E-18	2.409211927
Ano1	2.38E-18	3.039709984
Col1a2	5.02E-13	2.132928671
Cav1	5.10E-18	2.235452113
Bicc1	2.27E-19	2.486459956
Msln	2.26E-14	1.829701191
Dlc1	1.57E-18	2.561801087
Upk1b	1.96E-17	1.970013811
Sulf1	3.39E-17	2.542517734
Vim	1.68E-16	2.45290991
Sema3c	1.17E-17	2.547300887
Ifitm3	6.57E-15	2.071790767
Rspo1	1.96E-17	1.964755911
Aldh1a2	4.16E-15	2.160694165
Serping1	4.91E-14	2.101997058
Timp2	8.53E-10	1.596556868
Sdc4	9.47E-13	1.640281568
Cd34	2.04E-10	1.324668078
Serpinh1	2.72E-11	1.617681506
Heg1	1.56E-17	1.795303412
Arhgap18	1.69E-15	2.494302228
Igfbp7	3.27E-15	2.01497489
Wdr17	9.82E-15	3.265554301
Anxa2	1.55E-16	1.783316817
Celf2	8.01E-17	2.016354207
Slc4a4	2.64E-14	2.058956296
Mast4	7.11E-16	2.591248418
Bcam	3.45E-09	1.453472527
Cobl	3.19E-14	2.113252622
Airn	1.00E-16	2.159009448
Rbbp8	1.25E-07	1.460802144
Piezo2	6.47E-15	2.155464659
Wls	8.02E-16	1.68899014
Pcolce	2.35E-09	1.299424275
Prkg1	6.92E-15	2.705845188
Syne2	1.79E-15	2.085470282
Zfpm2	7.87E-15	2.689571135
Adamtsl1	1.19E-15	2.937092165
Gm26809	0.0001518	1.800107543
Chst4	1.09E-13	1.534679673
Ctdspl	1.02E-13	1.587097481
Ptgis	1.96E-10	1.436656186
Kcnab1	4.33E-13	2.347466136
Nav2	2.26E-14	2.852022736
C1ra	6.88E-07	1.318506858
Wnt5a	4.94E-12	1.540387353
Dab2	2.15E-13	1.86057873
Ankrd12	0.0001106	1.841901593
Cavin2	6.09E-12	1.976778556
Rnase4	1.39E-11	1.688218465
Meis1	1.26E-13	1.908870378
Anxa1	1.86E-12	1.531275304
Ildr2	9.47E-13	1.550620875
Slc9a3r1	1.86E-10	1.325162783
9530026P05	2.17E-13	2.515612487
Man1a	3.13E-12	1.539400492
Gpc3	1.20E-12	1.790707073
Col14a1	3.60E-11	2.039467045

MENs Cluster (Our Data)

Gene	FDR	summary.logFC
Bcam	0	1.310889756
Mgst1	0	1.466554292
Krt18	1.02E-252	0.722876212
Igfbp6	0	4.277079428
Rspo1	0	1.356888212
Gpm6a	0	2.007236002
Upk3b	0	2.136663175
Aebp1	0	2.502295384
Dcn	0	4.082052904
Slpi	0	4.928306229
Rarres2	0	4.202866323
Efemp1	0	1.672848073
Aldh1a2	4.60E-277	0.796263425
Fmo2	0	1.582315372
Upk1b	0	1.081912196
Gas1	0	1.464482623
Krt7	0	1.315380677
Crip1	0	4.56883561
Clu	0	1.502811937
C3	0	3.494711523
Dpp4	1.12E-210	0.556370013
Lrrn4	1.58E-271	0.736486954
Nkain4	0	1.298360281
S100a6	0	4.128650316
Krt19	0	1.900927424
Cfb	8.74E-203	0.608707666
Gabarapl1	5.26E-250	0.709728291
Lgals3	0	1.26072921
Krt8	1.39E-245	0.689105423
Tm4sf1	0	1.367716307
Slc39a8	7.17E-197	0.489802511
Sema3c	2.63E-255	0.71554962
Pcolce	0	1.823133603
Gsta4	1.09E-226	0.62733526
Ezr	0	1.019712533
Cldn15	4.76E-291	0.798661223
Serpinb6b	2.66E-212	0.562587863
Nbl1	0	1.239754358
Slc16a1	2.28E-244	0.65901005
Smim1	2.76E-245	0.659125894
Snhg18	3.11E-181	0.475288967
Cavin3	6.15E-293	0.855731534
Gpc3	0	1.393181642
Serpinb1a	3.54E-178	0.472277374
Chst4	3.42E-232	0.599828027
Esam	1.50E-188	0.47512383
Ptgis	5.59E-231	0.598750529
Slc9a3r1	7.03E-265	0.883988091
Ecm1	1.88E-172	0.514360839
Igfbp7	0	2.347561986
Msln	0	2.266588157
Gpx3	0	2.209730215
Col14a1	1.17E-163	0.423875564
Anxa1	4.24E-246	0.691007825
Bace2	7.94E-223	0.603024779
Cfh	0	1.190621234
Tmem176a	1.85E-170	0.915766916
Tmsb10	0	2.299200297
Timp2	0	1.918063461
Pdpn	2.36E-252	0.846386263
Prss23	0	0.980677037
Cd151	2.85E-279	0.795276372
P3h4	8.94E-212	0.512747523
Cd200	0	0.975177086
Ltbp4	1.80E-268	0.859534481
Gstt1	2.26E-142	0.347320974
Ssbp3	1.429035064	0.888557965
Tmem98	3.90E-240	0.62900078
Spint2	1.42E-186	0.478143896
Nupr1	3.42E-226	0.705142527
Basp1	6.06E-239	0.625848924
C1ra	2.06E-263	0.752700756
Serpinh1	4.940656458	1.065045464
Adamts2	9.08E-129	0.322228993
Dmkn	3.43E-187	0.652807485
C1s1	2.25E-276	0.820741368
Sparc	0	1.954167099
Espn	3.75E-166	0.392816171
Anxa2	0	1.579365041
Krt14	6.29E-117	0.368125408
Sdc4	0	1.913582811
Serping1	0	1.802367854
Pmp22	5.43E-232	0.6401978
Cplx2	1.77E-251	0.917909984
Il18	6.70E-154	0.396176068
Il6st	1.73E-262	0.71225818
Meg3	1.02E-173	0.558817278
Cald1	2.88E-262	0.803200156
Mt2	1.37E-218	0.736087263
Nenf	7.12E-165	0.869917467
Col6a1	2.61E-252	0.693856752
Rnase4	0.00E+00	1.162670662
Vim	0	1.805718628
F11r	6.92E-219	0.535570277
Fam180a	2.29E-131	0.294690768
Npdc1	2.34E-164	0.415479342
Cd2ap	2.16E-225	0.595177252
Pamr1	3.15E-177	0.443000918
Stk26	4.71E-112	0.258746973

Cluster annotated as Mesothelial (Drokhlyansky et al data)

Gene	FDR	summary.logFC
Lrrn4	5.4657E-206	16.886354
Rspo1	3.6499E-210	9.4753895
Wt1	1.5809E-227	10.114356
Upk3b	5.8625E-282	8.9449835
Bnc1	4.04195E-85	15.045354
Wt1os	2.3697E-123	9.9661905
Upk1b	1.277E-215	8.0787953
Aldh1a2	1.5275E-143	6.3791433
Msln	8.3474E-159	7.3877455
Igfbp6	1.7674E-307	7.7565747
Lvrn	4.18762E-92	7.8098721
C3	0	8.057149
Muc16	7.8925E-269	7.3116341
Gpm6a	0	6.5940179
Cybrd1	4.39858E-60	9.0017763
Fam180a	1.90191E-30	13.526168
Myrf	1.71228E-56	8.6635402
Aebp1	4.5764E-247	6.6271763
Crb2	2.91628E-52	7.3719161
Zdbf2	3.25895E-75	7.0872463
Sult5a1	2.85305E-24	13.31773
Cfh	4.6319E-250	5.7592104
Wdr17	3.1501E-282	6.6320991
Ildr2	3.4601E-136	6.7198792
Slpi	1.7684E-135	7.8326894
Fmod	2.27977E-40	8.7786829
Cpxm1	7.29311E-19	12.558464
Ifi205	2.62948E-18	12.864742
A730046J19	8.01459E-17	12.793582
Eya4	4.5788E-136	6.8974953
Nkain4	1.08326E-83	5.7593624
Col8a2	5.81471E-44	5.2884497
Zfp185	1.12186E-43	7.3412329
Dcn	6.4597E-292	5.6187432
Gm20400	7.4045E-135	7.0357895
Gas1	2.6756E-182	5.7763744
Cdh3	2.1841E-97	6.6406251
Efna5	1.6397E-264	4.7533052
Tmem151a	4.7978E-88	6.2910937
Bnc2	5.1982E-260	5.1670469
Gm12381	7.0467E-102	7.6933315
Serpinb6b	1.16024E-44	5.3575251
Plxna4	1.3438E-213	5.1141234
Efemp1	6.3046E-195	5.3926198
Gm765	4.94209E-43	6.7967853
Fmo3	1.17753E-20	8.4299998
Fmo2	2.9871E-243	5.3187177
Prss12	3.97653E-61	5.4000171
Vwa3a	1.02294E-65	5.8157216
Abcb1b	1.7423E-115	6.470465
Rarres2	6.6617E-142	5.7715796
Lrp2	2.01232E-42	6.2276692
Cldn10	6.74497E-17	5.6397899
Spock2	1.9494E-138	5.1124954
Chrd	1.6918E-22	7.4139259
Ltbp3	1.94368E-88	5.07873
Pkhd1l1	4.889E-120	4.4235079
Svopl	4.52096E-42	6.0169611
Ptgis	1.487E-109	5.0355511
Il16	1.59107E-97	4.7690361
Wnt9a	1.72493E-27	4.850231
Gabra3	2.92934E-57	4.859601
Medag	2.3907E-99	4.7656656
Atp6v0a4	1.46614E-75	5.0974539
Rpp25	1.286E-19	5.4541715
Bcam	2.8668E-131	5.0323969
Sulf1	4.0825E-179	4.5936014
Evc2	1.99898E-30	5.6828103
Arhgap28	6.6071E-111	4.5878415
Adamtsl1	6.7922E-140	4.3437642
Fam184a	3.53509E-66	5.6445631
Nxph1	9.70539E-83	6.4656245
Wnt5a	5.8364E-101	4.8191031
Unc13c	7.65838E-77	5.674392
Smtnl2	1.59692E-27	4.2520936
C1s1	7.3878E-125	4.5487014
Gata5	1.77132E-31	5.1168633
Tm4sf1	4.496E-113	4.5843519
Anxa9	9.60666E-21	4.8493462
Gpc3	4.9665E-99	4.7995673
Trpv4	7.95066E-16	5.3205717
Slit3	1.5771E-163	3.7434359
Synpr	1.67047E-53	5.2835493
Krt14	7.46374E-25	5.109502
Ifi204	4.97213E-20	5.7099144
Isl1	1.73444E-33	5.327459
Dnajc6	1.50196E-72	4.1858858
Meis1	1.3366E-165	3.8477795
Nnmt	6.20949E-19	5.3840664
Meis2	1.5618E-191	3.1626199
Cd34	1.84466E-86	3.7841694
Gm20186	1.72742E-38	4.7291369
Npr1	4.88291E-40	4.4804827
Gm11747	8.57184E-23	4.9058649
Mmp16	1.46268E-89	5.0348242
Loxl1	1.00489E-79	4.2443546
Ar	6.54875E-96	4.7109788
Rab7b	3.82339E-37	4.627583
Tnfrsf11b	1.46976E-58	5.0928081
Ccdc85a	1.53629E-69	4.4553075
