# Supplementary material for: Age-associated changes in lineage composition of the enteric nervous system regulate gut health and disease
Source: eLife. 2023 Dec 18;12:RP88051. doi: 10.7554/eLife.88051 (PMC10727506; doi:10.7554/eLife.88051)
Supplement: Supplementary file 1. [file elife-88051-supp1.pdf]

Supplementary Table 1: Table of information on select reagents

Primary Antibodies and Antisera

Antigenic target	Species	Company	Catalogue number	Dilution
HuC/D	Human	Human antisera ANNA1	ANNA1	1:750 - 1:1000
HuC/D	Rabbit	Abcam	ab184267	1: 500
NOS1	Rabbit	Invitrogen	61-7000	1: 200
CGRP	Rabbit	Immunostar	24112	1: 500
MET	Goat	R&D systems	AF276	1: 250
MHCst (S46)	Mouse	DHSB Iowa	S46	1: 250
RET	Rabbit	Abcam	ab134100	1: 500
CDH-3	Rabbit	Abcam	ab137729	1: 500
RFP	Rabbit	Rockland	600-401-379	1: 500
GFAP	Rabbit	DAKO	GA-524	1: 1000
β ACTIN	Mouse	MP Biomedicals	0869100-CF	1: 10000
HGF	Rabbit	Abcam	ab178395	1: 500
GDNF	Rabbit	Abcam	ab18956	1: 500
GFP	Chicken	Aves	GFP-1020	1: 1000
SLPI	Rabbit	Abbexa	abx104088	1: 200
AEBP1	Rabbit	Biorbyt	orb537407	1: 250
CLIC3	Rabbit	Proteintech	15971-1-AP	1: 250
CFTR	Rabbit	Abcam	ab181782	1: 500
SMO	Rabbit	Proteintech	20787-1-AP	1: 250
FMO2	Rabbit	Biorbyt	orb539506	1: 250
MYH11	Mouse	Thermo-Fisher	MA5-11971	1:250
SLC17A9	Rabbit	Proteintech	26731-1-AP	1:200
IL-18	Rabbit	Abcam	ab207323	1:400
NT-3	Rabbit	Chemicon	ab1532SP	1:200
MYL-7	Rabbit	Abcam	ab127001	1:100
SNAP-25	Rabbit	ProteinTech	14903-1-AP	1:200
PDE10A	Rabbit	Invitrogen	PA531293	1:250
TUBB2B	Rabbit	Invitrogen	PA5-48314	1:250
ELAVL2	Rabbit	Invitrogen	14008-1-AP	1:250
HAND2	Rabbit	Invitrogen	PA5-115339	1:250
VSNL1	Mouse	Invitrogen	67134-1-Ig	1:200
GPR88	Rabbit	Novus Biologicals	NBP1-02330	1:200
STMN2	Rabbit	Invitrogen	10586-1-AP	1:250
STX3	Rabbit	Invitrogen	15556-1-AP	1:250
ChAT	Rabbit	Abnova	PAB14536	1:100
DECORIN	Mouse	DSHB	6D6	1:250
ECT2	Rabbit	Bioss	BS-4102R	1:250
PHOX2B	Rabbit	Proteintech	25276-1-AP	1:250

Secondary Antibodies

Antigenic target	Species	Company	Catalogue number	Dilution
Anti-Rabbit 488	Goat	Invitrogen	A-11008	1:500
Anti-Rabbit 647	Goat	Invitrogen	A-21245	1:500
Anti-Rabbit 647	Donkey	Invitrogen	A-31573	1:500
Anti-Chicken 647	Donkey	Invitrogen	A-78952	1:500
Anti-Chicken 488	Donkey	Invitrogen	A-78948	1:500
Ant-Mouse 647	Donkey	Invitrogen	A-32787	1:500
Anti-Mouse IRdye 680RD	Donkey	Li-Cor	925-68072	1:10000
Anti-Rabbit IRdye 800CW	Donkey	Li-Cor	925-32213	1:10000

TaqMan Probes

Probe ID	Target	Company
Mm01135184_m1	Hgf	Invitrogen
Mm00599849_m1	Gdnf	Invitrogen
Mm00446968_m1	Hprt	Invitrogen
Mm00436304_m1	Ret	Invitrogen
