# Supplementary material for: Age-associated changes in lineage composition of the enteric nervous system regulate gut health and disease
Source: eLife. 2023 Dec 18;12:RP88051. doi: 10.7554/eLife.88051 (PMC10727506; doi:10.7554/eLife.88051)
Supplement: Supplementary file 3. [file elife-88051-supp3.pdf]

Supplementary File 3: Raw data of all experiments

Percentage of NC-derived and non-NC-derived neurons in adult Wnt1-cre:tdTomato mice

	No of neurons	% Wnt1:tdT+	% Wnt1:tdT-
**Mouse 1**	185	61.62	38.38
**Mouse 2**	276	53.62	46.38
**Mouse 3**	239	45.60	54.40
**Mouse 4**	485	54.64	45.36
**Mouse 5**	686	60.64	39.36
**Mouse 6**	345	58.26	41.74

Percentage of mesoderm-derived and non-mesoderm-derived neurons in adult Mesp1-cre:tdTomato mice

	No of neurons	% Mesp1:tdT+	% Mesp1:tdT-
**Mouse 1**	147	53.06	46.94
**Mouse 2**	137	53.28	46.72
**Mouse 3**	200	44.50	55.50

Percentage of tdTomato-expressing neurons of all NOS1+ neurons in adult Wnt1-cre:tdTomato mice

	NOS1+ counted	% Wnt1:tdT+	% Wnt1:tdT-
**Mouse 1**	248	64.04	35.96
**Mouse 2**	200	78.00	22.00
**Mouse 3**	168	76.78	23.22

Percentage of tdTomato-expressing neurons of all CGRP+ neurons in adult Wnt1-cre:tdTomato mice

	CGRP+ counted	% Wnt1:tdT+	% Wnt1:tdT-
**Mouse 1**	41	25.47	74.53
**Mouse 2**	56	23.37	76.63
**Mouse 3**	49	23.87	76.13

Age-wise changes in percentages of MENs in myenteric plexus neurons of Wnt1-cre:tdTomato mice

	**P11 mice**	
	**# neurons**	**% MENs**
**1**	374	2.14
**2**	534	10.00
**3**	419	0.23

	**P22 mice**		
	**# neurons**	**% MENs**	**Mean neurons/ganglia**
**1**	245	29.79	16.34 ± 2.07
**2**	175	27.43	11.67 ± 2.07
**3**	322	31.68	21.47 ± 1.61

	**P60 mice**		
	**# neurons**	**% MENs**	**Mean neurons/ganglia**
**1**	185	38.38	15.42 ± 3.59
**2**	276	46.38	21.23 ± 4.22
**3**	239	54.40	19.91 ± 3.26

	**P180 mice**		
	**# neurons**	**% MENs**	**Mean neurons/ganglia**
**1**	171	60.97	15.54 ± 2.52
**2**	239	50.39	21.73 ± 3.69
**3**	176	60.52	14.67 ± 1.80

	**P510 mice**		
	**# neurons**	**% MENs**	**Mean neurons/ganglia**
**1**	261	98.85	13.74 ± 2.22
**2**	360	93.22	17.14 ± 3.46
**3**	375	95.91	13.89 ± 1.30

Percentage of MENs in myenteric plexus of P20 Wnt1-cre:tdTomato mice dosed with and without GDNF from the P10 age

	**Control**		
	**# neurons**	**% MENs**	**Mean neurons/ganglia**
**1**	477	22.85	22.71 ± 6.99
**2**	296	20.27	12.86 ± 2.37
**3**	577	34.49	26.23 ± 4.55

	**GDNF**		
	**# neurons**	**% MENs**	**Mean neurons/ganglia**
**1**	418	3.83	19.90 ± 4.23
**2**	350	4.00	15.21 ± 3.38
**3**	533	3.75	38.00 ± 4.89

Percentage of MENs in myenteric plexus of P20 Wnt1-cre:tdTomato mice dosed with and without HGF from the P10 age

	**Control**		
	**# neurons**	**% MENs**	**Mean neurons/ganglia**
**1**	325	23.69	15.48 ± 1.88
**2**	439	17.77	20.90 ± 3.20
**3**	454	36.12	21.62 ± 3.40
**4**	336	44.05	16.00 ± 2.46
**5**	416	15.38	27.73 ± 3.73

	**HGF**		
	**# neurons**	**% MENs**	**Mean neurons/ganglia**
**1**	394	40.86	18.76 ± 2.51
**2**	409	40.83	19.48 ± 2.88
**3**	386	39.38	18.38 ± 2.71
**4**	326	62.27	15.52 ± 1.75
**5**	189	63.49	18.90 ± 2.01

Percentage of MENs in myenteric plexus of 17 month old mice dosed with and without GDNF

	**Control**		
	**# neurons**	**% MENs**	**Mean neurons/ganglia**
**1**	141	88.65	15.67 ± 5.22
**2**	238	93.28	21.64 ± 6.52
**3**	225	89.78	20.45 ± 6.16
**4**	213	84.04	18.54 ± 5.59
**5**	92	88.04	11.5 ± 4.06

	**GDNF**		
	**# neurons**	**% MENs**	**Mean neurons/ganglia**
**1**	111	84.68	12.33 ± 4.11
**2**	215	60.00	19.54 ± 5.89
**3**	160	88.12	14.54 ± 4.38
**4**	183	60.11	17.36 ± 5.23
**5**	130	77.69	16.25 ± 5.74

Percentage of RET+ NENs in myenteric plexus of 17 month old mice dosed with and without GDNF

	**Control**		
	**# neurons**	**% RET**	**Mean neurons/ganglia**
**1**	170	8.23	17.00 ± 5.37
**2**	227	6.17	20.64 ± 6.22
**3**	232	5.17	21.09 ± 6.36
**4**	188	9.04	18.8 ± 5.94

	**GDNF**		
	**# neurons**	**% RET**	**Mean neurons/ganglia**
**1**	137	19.70	13.70 ± 4.33
**2**	198	24.24	18.00 ± 5.43
**3**	200	11.00	18.18 ± 5.48
**4**	139	24.46	15.44 ± 5.14

Percentage of Ret-CFP+ NENs in myenteric plexus of Ret+/- mice at 9 and 16 weeks

	**9 weeks**		
	**# neurons**	**% CFP+**	**Mean neurons/ganglia**
**1**	268	14.18	24.36 ± 6.27
**2**	275	35.65	19.64 ± 2.28
**3**	294	28.91	24.50 ± 3.40

	**16 weeks**		
	**# neurons**	**% CFP+**	**Mean neurons/ganglia**
**1**	305	10.16	16.05 ± 3.68
**2**	306	13.40	13.91 ± 2.96
**3**	263	11.79	12.52 ± 2.73
**4**	122	15.57	11.09 ± 3.34
**5**	231	14.75	17.77 ± 4.93

Percentage of MHCst+ MENs in myenteric plexus of Ret+/- and Ret+/+ mice at 9 and 16 weeks

Ret+/+	**9 weeks**		
	**# neurons**	**% MENs**	**Mean neurons/ganglia**
**1**	197	41.12	17.91 ± 2.96
**2**	286	49.65	26.00 ± 3.96
**3**	297	39.06	19.80 ± 3.62

Ret+/+	**16 weeks**		
	**# neurons**	**% MENs**	**Mean neurons/ganglia**
**1**	217	56.68	19.73 ± 2.69
**2**	218	59.17	19.82 ± 3.12
**3**	316	60.44	19.75 ± 2.89
**4**	228	57.72	20.73 ± 4.45
**5**	297	38.38	18.56 ± 4.11

Ret+/-	**9 weeks**		
	**# neurons**	**% MENs**	**Mean neurons/ganglia**
**1**	220	44.54	20.00 ± 3.87
**2**	210	69.05	19.10 ± 2.73
**3**	214	62.62	13.37 ± 2.50

Ret+/-	**16 weeks**		
	**# neurons**	**% MENs**	**Mean neurons/ganglia**
**1**	182	91.21	16.54 ± 2.73
**2**	192	87.43	17.36 ± 1.25
**3**	215	83.25	13.43 ± 2.01
**4**	200	70.00	18.18 ± 3.74
**5**	147	82.84	21.00 ± 2.40

Percent MET+ neurons

	# MET+ neurons	Wnt1-tdT+	Wnt1-tdT-
**1**	68	16.18	83.82
**2**	56	17.86	82.14
**3**	28	21.43	78.57

Pattern Weights of the four MEN-specific NNMF patterns as represented in the post-natal mesenchymal single cell RNA sequencing data from Gut Cell Atlas

		**Pattern 16**			**Pattern 27**			**Pattern 32**			**Pattern 41**	
**Age**	Mean	Standard Error	Sample size	Mean	Standard Error	Sample size	Mean	Standard Error	Sample size	Mean	Standard Error	Sample size
Juvenile	0.0049	0.00023	5	-0.0036	0.0012	5	0.024	0.0005	5	-0.0029	0.0002	5
Adult	0.0049	0.0006	3	0.0085	0.0021	3	0.028	0.0030	3	0.0025	0.0028	3
Aging	0.0053	0.0007	3	0.0097	0.0019	3	0.033	0.0016	3	0.0058	0.0014	3

Age-wise changes in whole gut transit time of Ret+/+ and Ret+/- mice

**Age weeks**	**Whole gut transit time (in minutes) of *Ret* +/+ mice**								**Whole gut transit time (in minutes) of *Ret* +/- mice**									
**9**	131	119	59	135	113	88	103	86	73	105	93	91	87	83	134	143	153	132
**12**	83	89	89	100	106	86	110	78	89	124	106	98	77	71	128	73	82	124
**14**	107	103	120	141	111	117	166	110	100	189	190	93	107	106	190	207	125	96
**16**	111	130	106	116	130	122	116	140	200	237	194	122	185	157	102	101	156	119

Fold change (transcript expression) of Gdnf in murine small intestinal LM-MP at different ages

	P30	P90
**1**	0.8211	0.6884
**2**	0.6600	0.6081
**3**	1.6392	0.7047
**4**	1.6011	0.9263
**5**	0.7185	0.1380
**6**	0.9784	0.1683

Expression levels (Fluorescence intensity) of Hgf, compared to Beta-actin, in murine small intestinal LM-MP at different ages

	P10	P30	P90
**1**	0.008	0.007	0.022
**2**	0.006	0.017	0.013
**3**	0.006	0.011	0.016

Expression levels (Fluorescence intensity) of Gdnf, compared to Beta-actin, in murine small intestinal LM-MP at different ages

	P10	P30	P90
**1**	1.372	0.069	0.069
**2**	1.271	0.095	0.031
**3**	1.392	0.053	0.053

Whole gut transit time (in min) of 17 month old mice before and after GDNF or Saline treatment

	**Before Treatment**		**After Treatment**	
	**Control**	**GDNF**	**Control**	**GDNF**
1	210	182	210	74
2	210	210	164	110
3	210	152	168	87
4	172	210	162	123
5	210	210	171	111

Effect of GDNF on MHCst neuron proportions in 17 month old mice

	**Control**		**GDNF**	
	**Neurons counted**	**%MHCst**	**Neurons counted**	**%MHCst**
1	141	88.65	111	84.68
2	238	93.27	215	60.00
3	225	89.78	160	88.12
4	213	84.03	183	60.11
5	92	88.04	130	77.69

Effect of GDNF on RET neuron proportions in 17 month old mice

	**Control**		**GDNF**	
	**Fields counted**	**RET+**	**Fields counted**	**RET+**
1	10	14	10	27
2	10	14	10	48
3	10	12	10	22
4	12	17	12	34

Effect of GDNF on neuronal numbers in 17 month old mice

	**Control**	**GDNF**
**1**	15.67 ± 2.63	12.33 ± 3.55
**2**	21.64 ± 4.18	19.54 ± 2.55
**3**	20.45 ± 3.19	14.54 ± 1.91
**4**	18.54 ± 2.84	17.36 ± 2.61
**5**	11.50 ± 3.81	16.25 ± 2.30

Fold change (transcript expression) of Hgf in murine small intestinal LM-MP at different ages

	P10	P30	P90
**1**	2.146	5.442	6.114
**2**	1.867	4.747	5.337
**3**	0.543	2.919	4.745
**4**	0.659	2.683	4.123
**5**	0.789	2.703	5.593
**6**	0.884	2.928	5.824

Percent MHCst neurons that express tdTomato in Wnt1-cre:tdTomato mice

	MHCst+ neurons counted	Percent tdTomato+
**1**	29	0
**2**	30	0
**3**	48	0

UMI per cluster (6 month data)

	**Average ± SEM**	**# Cells**
**MENs**	4023.91 ± 85.35	2223
**NENs**	3391.71 ± 386.52	77
**Neuroglia**	1137.93 ± 27.93	1660

UMI per cluster (P21 data)

	**Average ± SEM**	**# Cells**
**MENs**	8891.84 ± 393.74	504
**NENs**	3263.34 ± 142.29	502
**Neuroglia**	3493.49 ± 120.25	835

Lengenivity Data

	**Mean and SEM**
**HGF**	0.0027 ± 0.0009
**GDNF**	-0.0008 ± 0.0004
**RET**	-0.0162 ± 0.0013

Percent ChAT+ neurons

	# neurons	Wnt1-tdT+	Wnt1-tdT-
**1**	440	68.77	31.23
**2**	594	74.84	25.16
**3**	648	77.31	22.69

Feret diameter (in microns)

	NENs	MENs
**1**	12.32 ± 0.34	17.38 ± 0.55
**2**	13.47 ± 0.42	18.38 ± 0.72
**3**	13.31 ± 0.44	16.65 ± 0.56

Ret transcripts in small intestinal LM-MP

	Ret WT	Ret Het
**1**	**0.777546**	**0.435879**
**2**	**1.232852**	**0.123450**
**3**	**1.043912**	**0.815478**
**4**		**0.583175**
