# Supplementary material for: Age-associated changes in lineage composition of the enteric nervous system regulate gut health and disease
Source: eLife. 2023 Dec 18;12:RP88051. doi: 10.7554/eLife.88051 (PMC10727506; doi:10.7554/eLife.88051)
Supplement: Supplementary file 4. [file elife-88051-supp4.pdf]

Supplementary File 4: scRNAseq data metrics for various detected subpopulations

scRNAseq data metrics from 6 month old mouse LM-MP cells

n_cells	annotated_type	pct_batch1	pct_batch2	num_genes	mt_ratio	umi
2248	Macrophage-A	0.551	0.449	238.8	0.09	440.98
2223	MENs	0.709	0.291	1255.92	0.05	4023.91
1736	Macrophage-B	0.548	0.452	1073.43	0.03	2654.48
1660	Neuroglia	0.74	0.26	633.96	0.06	1137.93
908	Vascular endothelium	0.792	0.208	854.27	0.07	1797.43
776	Smooth muscle cells	0.619	0.381	302.1	0.11	527.09
670	RBC	0.481	0.519	94.48	0.03	2380.12
240	Pdgfra+ Fibroblasts	0.542	0.458	776.06	0.06	1569.2
163	B Lymphocytes	0.067	0.933	602.87	0.05	1335.6
101	Penk+ Fibroblasts	0	1	1008.13	0.04	2272.25
80	T cells	0.125	0.875	763.98	0.05	1762.33
58	NENs	0.741	0.259	1031.98	0.07	3105.91
39	NK cells	0.256	0.744	752.21	0.04	1577.36
38	Macrophage-C	0.553	0.447	1631.5	0.03	4486.82
38	NK cells	0.184	0.816	711.11	0.04	1480
34	Macrophage-C	0.206	0.794	1334.94	0.02	4124.62
31	Smooth muscle cells B	0.742	0.258	588.87	0.05	975.58
22	Macrophage-B	0.727	0.273	1646.05	0.02	4178.14
22	Unknown	0.136	0.864	637.55	0.09	1617.18
19	NENs	0.105	0.895	1391.16	0.06	4264.16
17	Macrophage-C	0.294	0.706	1813.35	0.03	6721.53

scRNAseq data metrics from P21 mouse LM-MP cells

n_cells	annotated_type	pct_batch1	pct_batch2	num_genes	mt_ratio	umi	umi_sem
277	Endothelial	0.794	0.206	1712.19	0.05	5163.03	310.22
2165	Fibroblasts	0.824	0.176	1239.34	0.07	3187.54	68.51
312	ICC	0.76	0.24	1253.98	0.09	3001.8	169.94
231	Macrophage	0.879	0.121	2190.81	0.05	7270.13	478.34
510	MENs	0.751	0.249	1948.45	0.05	8891.84	393.74
526	NENs	0.743	0.257	1521.41	0.09	3263.34	142.29
844	Neuroglia	0.724	0.276	1501.25	0.06	3493.49	120.25
322	RBC	0.913	0.087	336.07	0.02	3792.37	211.34
5867	SMC	0.937	0.063	478.69	0.11	817.77	10.68
210	Unknown	0.91	0.09	1302.01	0.12	4767.06	328.96
